# Clear Cell Basal Cell Carcinoma

**DOI:** 10.4061/2011/386921

**Published:** 2011-04-20

**Authors:** Deba P. Sarma, Daniel Olson, Jennifer Olivella, Tracey Harbert, Bo Wang, Stephanie Ortman

**Affiliations:** ^1^Department of Pathology, Creighton University Medical Center, Omaha, NE 68131, USA; ^2^Department of Dermatology, Creighton University Medical Center, Omaha, NE 68131, USA

## Abstract

*Introduction*. Clear cell basal cell carcinoma (BCC) is an uncommon and unusual variant of BCC, which is characterized by a variable component of clear cells. The pathogenesis of this histological variant and its clinical significance has not been clarified. Differentiation of this uncommon variant of BCC from other clear cell tumors is important for the treatment. *Case Presentation*. A 65-year-old male presented with a 0.9 cm dome-shaped lesion on his upper chest. A shave biopsy revealed a dermal basaloid tumor that comprised nests with a peripheral palisading pattern, retraction artifacts, and striking clear cell changes. Histopathologic examination, along with findings from immunohistochemical studies and special staining of the clear cells, supports the diagnosis of clear cell basal cell carcinoma. *Conclusion*. Clear cell BCC is a rare and unusual variant of BCC. The underlying pathogenesis of this subtype is unclear; however, accurate identification may affect treatment and prognosis.

## 1. Introduction

Basal cell carcinoma is the most common cutaneous malignant tumor directly related to sunlight and UV radiation exposure. A number of histopathological subtypes of basal cell carcinoma have been defined. Among these subtypes, clear cell basal cell carcinoma is a rare variant composed of tumor cells with prominent cytoplasmic vacuoles or signet ring morphology. The pathogenesis of this variant, however, remains unclear. Distinguishing among basal cell variants as well as differentiating it from other clear cell tumors is important because treatment and prognosis of each may vary.

## 2. Case Presentation

**Figure 1:**
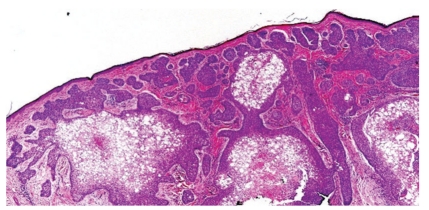
Low-power examination revealed a dermal basaloid tumor that comprised nests with clear cell changes present.

**Figure 2:**
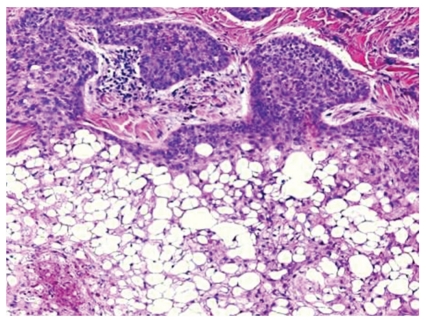
The clear cells are pleomorphic with a single large vacuole in their cytoplasm and a peripheral condensed hyperchromatic nucleus.

**Figure 3:**
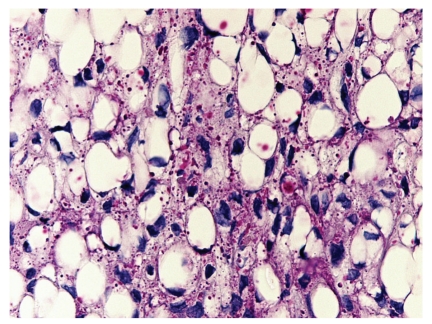
PAS without diastase stain shows positive cytoplasmic red material (glycogen) in the clear cells.

**Figure 4:**
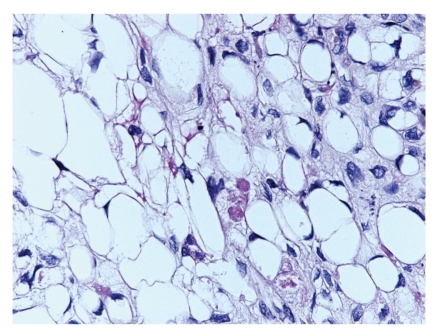
PAS with diastase stain is negative suggesting presence of glycogen but not mucin in the clear cells.

**Figure 5:**
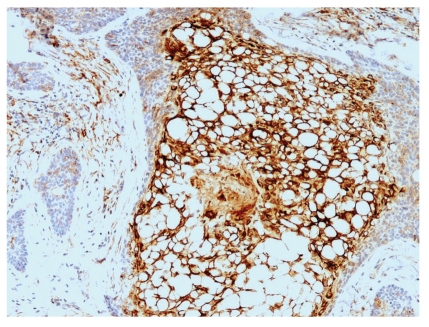
The clear cells are strongly positive with EMA immunostaining.

**Figure 6:**
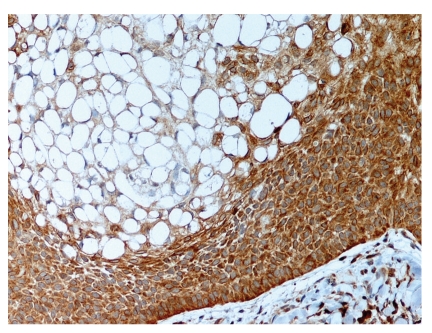
The peripheral basal cells are strongly positive with Bcl-2 protein immunostaining. The central clear cells are focally positive.

**Figure 7:**
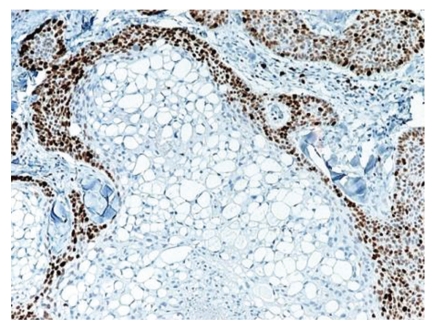
Ki-67 stain demonstrates negative nuclear staining in the clear cells in the center compared to the positive peripheral basal cells.

**Figure 8:**
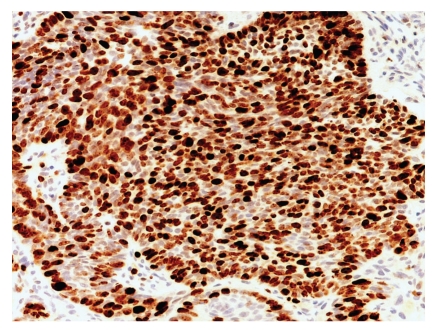
In contrast, a typical nodular basal cell carcinoma shows Ki-67-stained nuclei throughout the entire tumor nodule.

**Figure 9:**
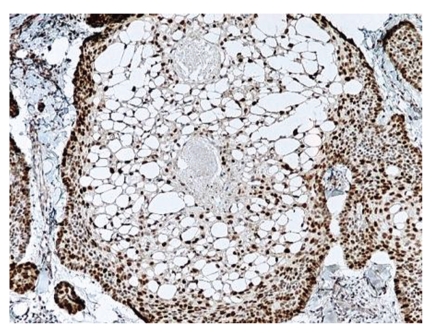
The basaloid cells and clear cells share a similar p53 nuclear mutation pattern.

A 65-year-old male presented with a 0.9 cm dome-shaped dark lesion on his upper chest. The clinical impression was basal cell carcinoma versus recurrent dysplastic nevus. The shave biopsy specimen showed a dermal tumor that comprised nests of basaloid cells with a peripheral palisading pattern and retraction artifacts (Figures 1 and 2). The basaloid cells were mitotically active, and apoptotic bodies were noted. The basaloid neoplasm manifested all features of ordinary basal cell carcinoma. However, striking clear cell changes were observed in the center of large nests (Figure 2). The clear cells were striking in appearance with a single large vacuole in their cytoplasm and a dark, condensed nucleus at the periphery. There were no clear cells with foamy-bubbly cytoplasm or starry nuclei; when present, this finding is typical of sebaceous cells. There were no clear pagetoid cells in the overlying epidermis. PAS staining was positive for the presence of glycogen (Figures 3 and 4), but no mucin was identified by mucicarmine stain. Immunohistochemical studies showed positive staining for EMA (Figure 5), Bcl-2 (Figure 6), CK5/6, and high molecular weight keratin but not for S 100 or vimentin. Ki-67 staining of the clear cells (Figure 7) was negative compared to the positive staining of a typical nodular basal cell carcinoma (Figure 8), suggesting a lack of proliferative activity. Additionally, no mitotic figures are seen on histopathologic examination of the clear cell components. The basaloid cells and the clear cells shared a similar p53 mutation pattern (Figure 9). Findings consistent with a clear cell basal cell carcinoma include morphology (typical nodular basal cell carcinoma pattern with central vacuolated cells without any foamy-bubbly cytoplasm), positive cytoplasmic glycogen, strongly positive Bcl-2 protein, positive CK 5/6, and positive high molecular weight keratin on immunostaining.

The lesion was completely excised with clear margins. Three years later, the patient remains free of any recurrence.

## 3. Discussion

Basal cell carcinoma (BCC) is the most common skin cancer, accounting for approximately 80% of nonmelanoma skin cancers. BCC usually occurs in sun exposed areas of the skin, most often the head and neck (80%), followed by the trunk (15%), and extremities [1]. Risk factors for developing BCC include exposure to ultraviolet radiation, a fair complexion, and immunosuppression. The clinical presentation can be quite variable, but lesions are typically described as shiny, pearly papules or nodules with a rolled border that may exhibit crusting, ulceration, or bleeding. 

BCC is thought to arise from pluripotential cells in the basal layer of the epidermis that are capable of differentiating into hair, sebaceous glands, or sweat glands. Perhaps as a consequence, there is considerable histopathologic variation among basal cell tumors, and several subtypes have been described. Sexton et al. reviewed 1039 cases of BCC and found the most common subtypes to be mixed (38.6%), nodular (21%), superficial (17.4%), and micronodular (14.5%) [2]. In addition, there are several rare variants, including basosquamous, morpheaform, keratotic, granular cell, adamantoid, and clear cell type. The utility in classifying BCC subtypes is that there appears to be a correlation between the histologic subtype and the clinical behavior. Micronodular, infiltrative, basosquamous, and morpheaform subtypes are more aggressive, while nodular and superficial types are less aggressive [3]. 

Clear cell basal cell carcinoma was first described by Barr and Williamson in 1984 [4]. A review of the literature to date reveals a total of 32 reported cases since that first description. Histochemical observations between cases have been inconsistent. PAS staining with and without diastase has been positive for glycogen in some cases [4–6] but not in others [7–10]. One report noted the presence of sialomucin deposition [6] constituting about 3% of the reported cases. Electron microscopic studies have also been variable, as some have observed the large intracellular vacuoles to be membrane bound [10, 11], while others have not [5, 7, 9]. 

 The differential diagnosis of clear cell basal cell carcinoma includes sebaceous carcinoma, clear cell squamous cell carcinoma, eccrine porocarcinoma, hidroadenocarcinoma, trichilemmal carcinoma, clear cell melanoma, and metastatic clear cell renal carcinoma. Sebaceous carcinoma cells with “foamy-bubbly” cytoplasm are positive for lipid on staining with Oil Red O or Sudan IV on frozen tissue and are positive with EMA and CAM 5.2 immunostaining. A small amount of cytoplasmic glycogen may be present. Clear cell squamous cell carcinoma is characterized by evidence of hydropic change of the neoplastic cells with accumulation of intracellular fluid and shows areas of squamous differentiation with foci of keratinization and keratin pearls. Unlike other cutaneous clear cell neoplasms, the accumulated fluid does not stain for glycogen, lipid, or mucin. The clear cells of eccrine porocarcinoma contain glycogen shown by diastase-resistant PAS positive cytoplasm. Moreover, they are positive with CEA and EMA immunostaining. The clear cells seen in eccrine hidroadenocarcinoma contain diastase-resistant PAS positive cytoplasmic glycogen. They are also positive with CEA and S-100 protein immunostaining. In contrast, clear cells in trichilemmal carcinoma have abundant glycogen but exhibit CEA and EMA negativity. The clear cell subtype of melanoma, also known as balloon cell melanoma, is usually S-100 and HMB-45 positive. Finally, the clear cells of metastatic renal cell carcinoma contain lipid (stained with Oil Red O) and glycogen but are negative for CEA and S-100 protein.

Because the clear cells of clear cell BCC stain positive for glycogen, the pathogenesis was initially suggested to be due to trichilemmal differentiation [4]. Subsequent hypothesis have suggested that the large, clear membrane-bound vacuoles are phagolysosomes containing degradation products of intracellular organelles [7, 10]. However, the underlying mechanisms responsible for this histologic subtype of BCC are unknown. 

## 4. Conclusion

Clear cell BCC is a rare and unusual variant of BCC. The underlying pathogenesis, clinical effect on treatment, and prognosis of this subtype remain unclear.
